# Does Seasonality Affect Peptic Ulcer Perforation? A Single-Center Retrospective Study

**DOI:** 10.3390/medicina61060945

**Published:** 2025-05-22

**Authors:** Iva Krajnović, Zenon Pogorelić, Iva Perić, Marija Ćavar, Matija Borić

**Affiliations:** 1 Department of Surgery, School of Medicine, University of Split, 21000 Split, Croatia; 2 Department of Pediatric Surgery, University Hospital of Split, 21000 Split, Croatia; 3 Clinical Department of Diagnostic and Interventional Radiology, University Hospital of Split, 21000 Split, Croatia; 4 Department of Surgery, University Hospital of Split, 21000 Split, Croatia

**Keywords:** peptic ulcer perforation, peptic ulcer disease, seasonal variation, risk factors, smoking, alcohol consumption, emergency surgery

## Abstract

*Background and Objectives:* Perforated peptic ulcers are a common surgical emergency and rank among the leading causes of acute peritonitis worldwide. Previous studies have suggested a seasonal pattern in the occurrence of symptomatic perforated peptic ulcers. With the advancement of modern medicine, including the widespread use of proton pump inhibitors, and the effects of climate change, this study aimed to assess potential seasonal variations in the incidence of peptic ulcer perforation in our region. *Methods:* This retrospective analysis included 104 adult patients (mean age: 61.5 ± 14.7 years) who underwent surgical treatment for peptic ulcer perforation between January 2021 and April 2024. Patients were analyzed by gender, age, risk factors (smoking and alcohol consumption), the location of the perforation (gastric or duodenal), and discharge outcome (survived or deceased). Additionally, cases were categorized by the month and season of the ulcer perforation. *Results*: Among the 104 patients (mean age 61.5 ± 14.7 years), 68 (65.4%) were male. Gastric and duodenal perforations were nearly equally observed (51% vs. 49%). A statistically significant difference in overall perforation rates by gender was observed (*p* = 0.009), though not between ulcer sites (*p* = 0.628 and *p* = 0.739). The highest number of perforations occurred in July (*n* = 12), while the lowest occurred in November (*n* = 4); however, no significant variation was found by month (*p* = 0.916) or season (*p* = 0.891), despite a predominance in spring. Comorbidities were present in 60% of patients. Smoking (33.6%) and alcohol use (22.1%) were common. Alcohol abuse was noted in 22.1% of patients and was significantly associated with both gastric (*p* < 0.001) and duodenal (*p* < 0.001) perforations, though not with the overall incidence (*p* = 0.284). Smoking, reported in 33.6% of patients, showed no significant association with the perforation site or overall incidence (*p* = 0.946). The combination of smoking and alcohol use favored gastric perforations, but without statistical significance (*p* = 0.157). *Conclusions*: Alcohol consumption appeared to increase the risk of ulcer perforation, while smoking did not demonstrate a statistically significant association. Although spring exhibited the highest observed incidence of peptic ulcer perforation, seasonal variation did not show a statistically significant difference overall.

## 1. Introduction

Peptic ulcer disease, which used to be treated almost exclusively by surgery, is now mainly treated using pharmacological therapy. This change resulted from significant advances in understanding the underlying mechanisms of the disease and the development of effective pharmaceutical treatments. Peptic ulcer disease occurs when there is an imbalance between protective mechanisms, such as the secretion of mucus and bicarbonate, adequate blood flow to the mucosa, and the integrity of the epithelium, and harmful factors that erode the gastric or duodenal mucosa. These damaging factors include excessive stomach acid production, infection with Helicobacter pylori, the use of non-steroidal anti-inflammatory drugs (NSAIDs), smoking, and excessive alcohol consumption. Together, these factors disrupt the natural defenses of the gastrointestinal mucosa and lead to the formation of ulcers [1].

The introduction of proton pump inhibitors (PPIs), which are highly effective in suppressing gastric acid production, together with antibiotic regimens aimed at eradicating *H. pylori*, has dramatically altered the course of peptic ulcer treatment. These advances have significantly reduced the prevalence of the disease, particularly in Western countries where PPIs are widely available and regularly used [2]. Modern diagnostic imaging techniques, such as contrast-enhanced radiography and CT scans, also play a role in the detection of complications of peptic ulcer disease, particularly in the case of perforation or obstruction. In terms of diagnosis, modern endoscopic procedures, such as upper gastrointestinal endoscopy, allow the more accurate identification of the type, location, and severity of the ulcer, greatly improving the ability to monitor disease progression. In addition, non-invasive tests for *H. pylori*, including urea breath tests and stool antigen tests, have improved diagnostic accuracy and patient convenience. The most serious complications include upper gastrointestinal bleeding, perforation, and gastric outlet obstruction. These conditions often require surgical treatment and are associated with significant morbidity and mortality [3,4]. The early detection of complications using these diagnostic methods significantly improves its prognosis and enables timely interventions. The prognosis can vary greatly depending on the time of diagnosis and the occurrence of complications. Early diagnosis and timely intervention, particularly for perforated ulcers, are crucial to improve outcomes and reduce the need for surgical intervention. Advances in diagnosis and treatment have contributed to a reduction in mortality rates related to peptic ulcer disease.

Among these complications, a perforated peptic ulcer is particularly critical. It remains one of the most common causes of acute peritonitis and is a frequent surgical emergency worldwide. This breach in the gastrointestinal barrier triggers a strong inflammatory response, leading to peritonitis [5]. Surgical repair remains the cornerstone of perforated peptic ulcer treatment. Traditionally, laparotomy with omental patch repair is the gold standard. This technique involves suturing a portion of the omentum over the perforation to reinforce closure and aid the healing process. However, with advances in surgical techniques and the increasing prevalence of minimally invasive procedures, laparoscopic repair has proven to be an effective alternative. Laparoscopy offers several advantages over traditional open surgery, including reduced post-operative pain, shorter hospital stays, faster recovery times, and better cosmetic results. These advantages make laparoscopy an increasingly popular option, especially in centers with experienced surgeons [6,7]. However, the decision between laparoscopic and open surgery often depends on the size of the perforation, the patient’s condition, and the surgeon’s experience. In cases where the ulcer is unusually large or a malignant ulcer is suspected, more extensive procedures such as partial gastric resection may be required to remove the affected tissue and ensure complete healing [8].

An interesting aspect of peptic ulcer disease, especially its symptomatic and complicated forms, is its possible seasonal variation. Historical data suggest that there may be seasonal peaks in the incidence of ulcer-related complications such as bleeding or perforation. However, the underlying causes of these patterns remain unclear, and there is no consensus in the medical community as to their significance or persistence. Several factors have been proposed to explain these variations, including seasonal changes in dietary habits, stress, and infection rates. Environmental variables such as temperature and humidity may also play a role in influencing gastric acid secretion or the integrity of the gastric mucosa [9].

The impact of climate change on these seasonal variations is a topic of growing interest. As global temperatures rise and seasonal patterns shift, the environmental factors that influence the development and progression of peptic ulcer disease could also change. For example, fluctuations in the transmission rates of *H. pylori*, changes in NSAID consumption patterns during certain seasons, and shifts in dietary behavior due to climatic factors could alter the epidemiology of peptic ulcers. Innovative diagnostic approaches such as genetic markers and biomarkers for *H. pylori* infections are also being researched and offer the potential for even more personalized diagnosis and treatment.

The aim of this study was to investigate whether seasonal patterns in the incidence of peptic ulcer perforation persist in the context of modern medical advances, including improved PPIs, and the ongoing effects of climate change. This research may help in understanding how seasonal variation continues to influence the diagnosis, prognosis, and management of peptic ulcer disease despite advancements in treatment. By identifying these patterns, healthcare providers may be able to implement targeted prevention strategies, optimize the timing of interventions, and anticipate fluctuations in need of medical care during high-risk periods. This research is important not only to improve our understanding of the disease but also to improve patient outcomes and ensure that medical resources are used effectively.

## 2. Methods

### 2.1. Patients

This retrospective cohort study included a total of 104 adult patients who underwent surgery for perforated peptic ulcers in the Department of Surgery at the University Hospital of Split between 1 January 2021 and 1 April 2024. The inclusion criteria were patients over 18 years of age who underwent surgery for perforated peptic ulcers in our institution. The exclusion criteria were patients under 18 years of age, patients with incomplete medical records, and patients with a perforation due to a histologically confirmed malignancy. In each patient with gastroduodenal ulcer perforation, surgical intervention was indicated after a clinical examination and diagnostic procedure, and an omental patch was performed using either an open or laparoscopic method.

### 2.2. Ethical Aspects

This study was carried out in strict adherence to the ethical principles outlined in the Declaration of Helsinki, a cornerstone document of the World Medical Association that provides guidelines for conducting medical research involving human participants. The Institutional Review Board of our hospital approved this study (approval number: 520-03/24-01/34; date of approval: 22 February 2024).

### 2.3. Study Design

The following parameters were recorded for each patient included in this study: demographic data (age, sex), date of surgery, site of perforation (stomach or duodenum), and concomitant diseases. In addition to the clinical and procedural details, this study also accounted for any concomitant diseases present in the patients, such as diabetes, hypertension, or cardiovascular conditions, as these could potentially affect the healing process or increase the risk of complications. The patients’ habits, particularly smoking and alcohol consumption, were carefully documented, as both of these habits are well-established risk factors for peptic ulcer disease and its complications, influencing ulcer formation, healing, and the likelihood of perforation. Smoking was defined as daily tobacco use, while alcohol consumption was defined as medium or high risk if the quantity ingested was more than 40 g/day for men and more than 20 g/day for women. Pathohistologic findings were also recorded, offering valuable information about the nature of each ulcer.

The seasons were categorized as follows: spring (21 March–20 June), summer (21 June–22 September), autumn (23 September–20 December), and winter (21 December–20 March). In our region (Split, Croatia), seasonal changes are accompanied by significant temperature fluctuations that can affect the gastrointestinal physiology and behavior of patients. Average temperatures range from 5 °C to 13 °C in winter, 12 °C to 21 °C in spring, 22 °C to 30 °C in summer, and 10 °C to 20 °C in autumn.

### 2.4. Outcomes of This Study

The primary outcome was to investigate the seasonal variations in peptic ulcer perforations in our cohort of patients. The secondary outcome was to assess the association between smoking habits or alcohol consumption and peptic ulcer perforation.

### 2.5. Statistical Analysis

Statistical data analyses were performed using MedCalc software (version 23.1.0, MedCalc Software Ltd., Ostend, Belgium, available at https://www.medcalc.org/, accessed on 19 June 2024). Data visualization was performed using Matplotlib (Version 3.7.1; Matplotlib Development Team, Austin, TX, USA). A *p*-value of less than 0.05 was considered statistically significant. The Kolmogorov–Smirnov test was used to determine whether the data were normally distributed. Given that all the variables in the dataset demonstrated a normal distribution, the mean ± standard deviation (SD) was used to describe the distribution of the quantitative data. For categorical variables, the distribution was expressed using absolute numbers and their corresponding percentages, while the Chi-square test was used for comparison. Fisher’s exact test was applied when expected cell counts were low.

## 3. Results

A total of 104 patients who underwent surgery for perforated peptic ulcers between 1 January 2021 and 1 January 2024 at our institution were included in this retrospective study. The mean age of the patients was 61.5 ± 14.7 years. In regard to gender, 68 of the patients were male (65.4%) and 36 were female (34.6%). Gastric perforation was found in 53 (51%) patients, and duodenal perforation in 51 (49%) patients. A statistically significant difference in the peptic ulcer perforations by gender was recorded (Table 1).

**Figure 1:**
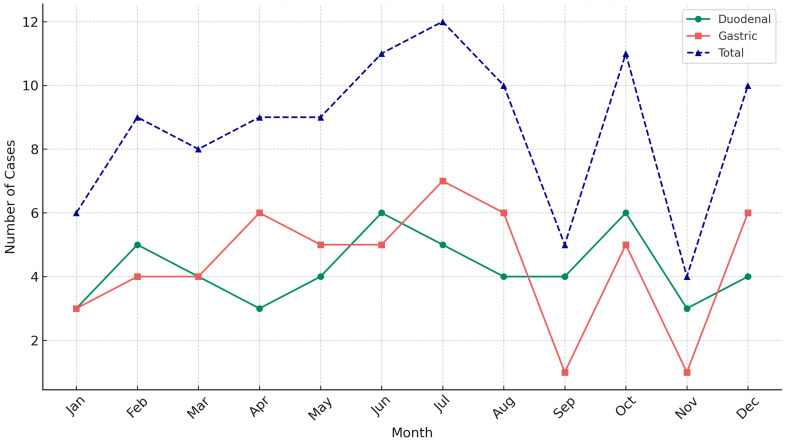
Monthly distribution of peptic ulcer perforation cases. Green line—duodenal perforations; red line—gastric perforations; blue line—total rate of perforations.

The primary aim of our study was to determine whether there was a seasonal or monthly difference in the incidence of perforated peptic ulcers. The highest case count was found in July, and the lowest in November (Table 2, Figure 1).

**Figure 2:**
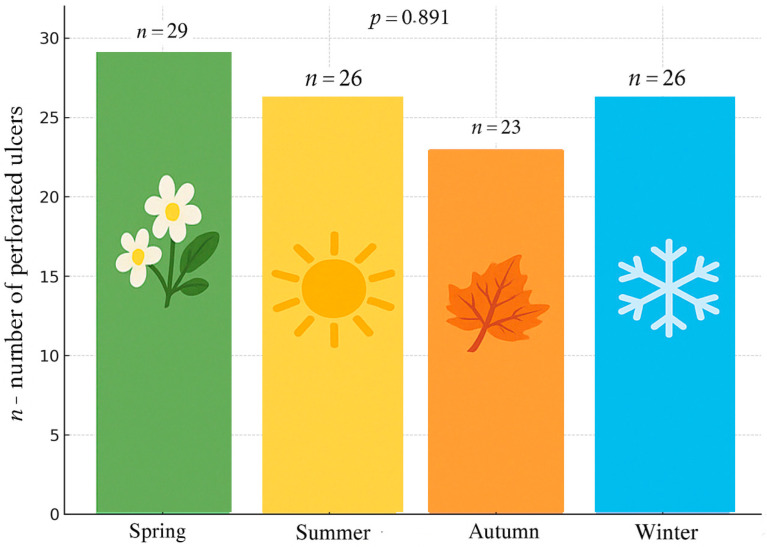
Seasonal variations in peptic ulcer perforations (*n* = 104; *p* = 0.891).

When comparing the incidence by season, peptic ulcer perforation was least common in the autumn (Table 3, Figure 2).

Comorbidity was assessed for patients in terms of hypertension (HT), diabetes mellitus (DM), coronary artery disease, congestive heart failure, chronic obstructive pulmonary disease, and others. In total, 62 patients (60%) had comorbidities, 35 patients (34%) had HT, and 9 patients (9%) had DM.

An analysis of the patient’s habits revealed that 18 (17.3%) both consumed alcohol and smoked. Of these 18 individuals, 12 (66.7%) had a gastric ulcer, while 6 (33.3%) had a duodenal ulcer. A majority, specifically 61 (58.7%) of the included patients, neither smoked nor consumed alcohol in significant amounts, and of these, 31 (50.8%) had a duodenal ulcer and 30 (49.2%) had a gastric ulcer. Only 17 (16.4%) were smokers, of whom 11 (64.7%) had a duodenal ulcer and 6 (35.3%) had a gastric ulcer. Of the total number of respondents, five (4.8%) consumed only alcohol, and of these, three (60%) suffered from duodenal ulcers and two (40%) from gastric ulcers. Despite these assumptions, we were unable to detect a statistically significant difference in this patient sample that would indicate a combination of smoking and alcohol consumption as a negative lifestyle habit that would further increase the risk of ulcer perforation (Table 4).

Out of the 104 patients, smoking as a risk factor was recorded in 35 patients (33.6%). No statistically significant difference in the perforation rate between smokers and non-smokers was found (Table 5).

Alcohol abuse was found in 23 patients (22.1%). There was a statistically significant difference in the amount of alcohol abuse between the duodenal and gastric perforation groups, with no difference in the overall number of patients (Table 6).

## 4. Discussion

Meteorological and climatic parameters have previously been associated with numerous health disorders [10]. Air temperature could cause an imbalance between gastric acid secretion and the defense system of the gastric mucosa, leading to gastric ulcers [11,12]. Although the pathophysiological mechanism of these seasonal fluctuations remains unclear, it could be explained by the interaction of climate changes, medication, or *H. pylori* infection [13,14]. In addition, increased alcohol consumption during the cold months could partially explain older observations. Although there are many previous studies on seasonal variations in peptic ulcer perforation, their results are limited due to the small sample size of these studies, and since these studies have been conducted worldwide, there has not been one unified conclusion so far [15,16,17,18]. In the last two decades, only the study by Manfredini et al. was carried out in a climate region similar to ours [9].

Despite the lack of a definitive pathophysiologic explanation, the association between the climate and peptic ulcer perforation underscores the importance of considering environmental factors in the care and treatment of patients with peptic ulcers. Variations in climatic parameters such as temperature, humidity, and air pressure could directly or indirectly influence physiologic stress responses, gastric acid secretion, and the integrity of the gastric mucosa. Seasonal changes in lifestyle, including dietary and activity patterns, could also contribute to the observed differences. These findings underscore the need for a multidisciplinary approach that incorporates meteorological data and behavioral trends into public health strategies to prevent gastrointestinal disease.

More recent studies have shown an increase in the proportion of women with perforated ulcers [19]. Our study is consistent with these observations. In addition, our study confirmed the predominance of men [20]. Seasonal trends in peptic ulcer disease exacerbation represent a debatable issue. Previous studies have focused on different aspects of peptic ulcer disease, from uncomplicated ones to those with complications such as bleeding or perforation. The conflicting results regarding seasonal trends in peptic ulcer perforation could be explained by a limited sample size or a wide range of different geographical distributions of the observed populations [21,22,23,24]. We observed the highest incidence of peptic ulcer perforation in July and the lowest in November. Both too-hot and too-cold weather could lead to the stress-induced exacerbation of peptic ulcers. As there are no extreme winter temperatures in our region, this could lead to the incidence peak in July.

The peak in July observed in our study raises interesting questions about the role of warm weather in exacerbating stress-related physiological responses. Studies suggest that high temperatures can lead to dehydration, which in turn would reduce blood flow to the stomach lining and impair its defenses. In addition, heat-related dietary habits, such as an increased consumption of spicy or preserved foods, may also play a role in exacerbating the formation of ulcers or perforations. This link between high temperatures and gastric health needs further investigation to confirm the cause and clarify the mechanisms involved.

The relationship between peptic ulcer perforation and the season is not well established. The seasonal trends in gastric ulcer hospitalizations are still debated. Some studies report a peak in winter and a trough in summer [16,25]. A large study from the United States reported a peak in spring and a decline in autumn [26]. In addition, the data from the literature are controversial, as some studies show a peak in winter, some in late summer, and some at the end of spring and autumn [18,24,27]. In one study, the incidence of hematemesis due to a peptic ulcer was found to be inversely proportional to temperature and relative humidity and proportional to barometric pressure throughout the year [28]. In another study, a close correlation was found between the incidence of peptic ulcers and mean temperature, mean maximum and minimum temperatures, mean air pressure, and mean dew point temperature [9]. A recent study found that hospitalizations for duodenal ulcers were related to temperature. In particular, there was a peak in winter and a decline in summer in patients aged 35 to 49 years and over 50 years [29].

These differences highlight the complexity of environmental influences on peptic ulcers. While cold weather is often associated with physiological stress due to increased sympathetic nervous system activity, which may compromise gastric mucosal defenses, warm weather may similarly affect gastric physiology through dehydration or increased oxidative stress. It is possible that individual susceptibility, including genetic predispositions, comorbidities, and behavioral factors such as diet and alcohol consumption, may modulate the seasonal impact of these environmental parameters. Such multifactorial considerations could explain why existing studies report divergent seasonal patterns.

In the colder months, the human body experiences significant acute stress due to the cold and constant temperature fluctuations, caused by the stimulation of the sympathetic nervous system and the rapid release of norepinephrine and adrenaline, which leads to the contraction of the blood vessels of the duodenal mucosa, ultimately resulting in damage to the mucosa due to insufficient oxygen supply. In addition to meteorological factors, the seasonal variations observed in this study could also be because most people with conditions such as osteoarthritis or rheumatoid arthritis worsen during the winter months, leading to an increased use of non-steroidal anti-inflammatory drugs [30].

The interaction between cold weather and medication use is an important area for future research. NSAIDs are well-documented risk factors for peptic ulcers, but their seasonal patterns of use and cumulative effects have not been adequately explored. Strategies to mitigate the seasonal increase in NSAID-related complications could include patient education, alternative pain management methods, and increased surveillance during the colder months. In addition, targeted therapeutic interventions to address stress and vascular changes in winter could help reduce the burden of peptic ulcer complications.

It is known that alcohol abuse can damage the stomach or duodenum by compromising the integrity of the mucosal barrier. Oxidative stress is involved in the pathogenesis of ethanol-induced gastric mucosal damage, leading to hemorrhagic injury or perforation [31]. Excessive and regular alcohol consumption and smoking are two risk factors that may be associated with the development of peptic ulcers. Inflammatory changes found in patients with chronic gastritis are associated with concomitant Helicobacter pylori infection, which is common in alcoholics. Long-term alcohol consumption is associated with gastric metaplasia, alterations in the histology of the mucosa, and the disruption of the mucosal barrier. These changes and direct damage play a key role in disrupting and altering the defense mechanisms of the gastric mucosa [32]. In the present study, a statistically significant difference in the incidence of ulcers was found as a function of alcohol consumption. Of the patients who consumed alcohol, the majority had stomach ulcers.

This association between alcohol consumption and peptic ulcer perforation may be due to a complex interaction of biochemical, microbial, and lifestyle factors. The role of alcohol-induced oxidative stress in exacerbating mucosal injury underscores the need for antioxidant-based therapeutic strategies in high-risk populations. Furthermore, public health measures to address excessive alcohol consumption, particularly during seasons of heightened stress or social drinking patterns, could play a significant role in reducing the incidence of peptic ulcer complications.

The seasonal habits of alcohol consumption and its effects on peptic ulcer perforation need further study. Researchers have found that cigarette smoking is the most common risk factor for the increased incidence of perforated ulcers [33,34]. Tobacco is thought to inhibit pancreatic bicarbonate secretion, leading to increased acidity in the duodenum [5]. In contrast to alcohol consumption, this study found no statistically significant difference in the incidence of perforations between smokers and non-smokers, which is not in line with recent studies [35].

While our results regarding smoking and ulcer perforation differ from the existing literature, they emphasize the variability in individual responses to smoking-induced gastric changes. Differences in study populations, smoking habits, or other environmental influences may explain these discrepancies. Future studies should consider the combined effects of smoking, alcohol consumption, and other risk factors, as their synergistic effects could provide deeper insights into the pathophysiology of peptic ulcers.

This study was limited primarily by its small sample size, as it was a single-center study; in addition, retrospective data limited the accuracy of the results due to missing information. In addition, our study did not include data on *H. pylori* infection or NSAID use—both important risk factors for peptic ulcer disease and its complications. This limited our ability to fully assess the etiological backgrounds of the perforations. We also did not consider potential seasonal variations in healthcare-seeking behavior, which may have influenced the observed incidence patterns. Future studies should include these variables to provide a more comprehensive understanding.

Despite these limitations, our study provides valuable insights into seasonal and behavioral influences on peptic ulcer perforation. Expanding this research to larger, multicenter cohorts using advanced data collection methods, such as wearable sensors to monitor stress and physiological changes, could provide a more comprehensive understanding of this multifactorial disease. The integration of environmental data with patient-specific factors in predictive modeling could pave the way for personalized approaches to peptic ulcer prevention and management.

## 5. Conclusions

The highest level of peptic ulcer perforation was recorded in July, and the lowest was in November. In an analysis of the data by season, the perforation of peptic ulcers was lowest in the fall. Alcohol abuse was found to be a statistically significant risk factor in both the duodenal and gastric perforation groups. Further analyses of seasonal differences in alcohol or smoking habits should be conducted in a larger population.

## Figures and Tables

**Table 1:** Comparison of ulcer perforation rates with the gender of the patient.

Gender	Duodenum *n* (%)	Stomach *n* (%)	Total *n* (%)	*p* *
Male	32 (30.8)	36 (34.6)	68 (65.4)	0.628
Female	19 (18.3)	17 (16.3)	36 (34.6)	0.739
Total	51 (49)	53 (51)	104 (100)	0.009

* Chi-square test.

**Table 2:** Number of cases of peptic ulcer perforation by month.

Month	Duodenum *n* (%)	Stomach *n* (%)	Total *n* (%)	*p* *
January	3 (2.9)	3 (2.9)	6 (5.8)	1.000
February	5 (4.8)	4 (3.8)	9 (8.7)	0.739
March	4 (3.8)	4 (3.8)	8 (7.7)	1.000
April	3 (2.9)	6 (5.8)	9 (8.7)	0.317
May	4 (3.8)	5 (4.8)	9 (8.7)	0.739
June	6 (5.8)	5 (4.8)	11 (10.6)	0.763
July	5 (4.8)	7 (6.7)	12 (11.5)	0.564
August	4 (3.8)	6 (5.8)	10 (9.6)	0.527
September	4 (3.8)	1 (1.0)	5 (4.8)	0.180
October	6 (5.8)	5 (4.8)	11 (10.6)	0.763
November	3 (2.9)	1 (1.0)	4 (3.8)	0.317
December	4 (3.8)	6 (5.8)	10 (9.6)	0.527
Total	51 (49)	53 (51)	104 (100)	*p* = 0.916

* Chi-square test.

**Table 3:** Number of cases of peptic ulcer perforation by season.

Season	Duodenum *n* (%)	Stomach *n* (%)	Total *n* (%)	*p* *
Spring	13 (12.5)	16 (16.4)	29 (27.9)	0.578
Summer	12 (11.5)	14 (13.5)	26 (25.0)	0.695
Autumn	12 (11.5)	11 (10.6)	23 (22.1)	0.835
Winter	14 (13.5)	12 (11.5)	26 (25.0)	0.695
	*p* = 0.975	*p* = 0.801	*p* = 0.891	

* Chi-square test.

**Table 4:** Distribution of peptic ulcer perforation according to risk factors.

Risk Factors	Duodenum *n*	Stomach *n*	Total *n*	*p* *
None	31 (29.8)	30 (28.8)	61 (58.7)	0.695
Alcohol	3 (2.9)	2 (1.9)	5 (4.8)	0.675
Smoking	11 (10.6)	6 (5.8)	17 (16.4)	0.191
Alcohol and smoking	6 (5.8)	12 (11.5)	18 (17.3)	0.196
	*p* < 0.001	*p* < 0.001	*p* = 0.297	

* Fisher’s exact test.

**Table 5:** Distribution of peptic ulcer perforations according to the site of perforation and smoking.

Smoking	Duodenum *n*	Stomach *n*	Total *n*	*p* *
Yes	17 (16.3)	18 (17.3)	35 (33.6)	0.866
No	34 (32.7)	35 (33.7)	69 (66.4)	0.904
	*p* = 0.017	*p* = 0.0195	*p* = 0.946	

* Chi-square test.

**Table 6:** Distribution of peptic ulcer perforations according to the site of perforation and alcohol abuse.

Alcohol Abuse	Duodenum *n*	Stomach *n*	Total *n*	*p* *
Yes	9 (8.7)	14 (13.5)	23 (22.1)	0.297
No	42 (40.4)	39 (37.5)	81 (77.9)	0.739
	*p* < 0.001	*p* < 0.001	*p* = 0.284	

* Chi-square test.

## Data Availability

The data presented in this study are available on request from the corresponding author.

## References

[1] Coco D., Leanza S. (2022). A Review on Treatment of Perforated Peptic Ulcer by Minimally Invasive Techniques. Maedica.

[2] Costa G., Fransvea P., Lepre L., Liotta G., Mazzoni G., Biloslavo A., Bianchi V., Occhionorelli S., Costa A., Sganga G. (2023). Perforated peptic ulcer (PPU) treatment: An Italian nationwide propensity score-matched cohort study investigating laparoscopic vs open approach. Surg. Endosc..

[3] Vidović S., Borović S., Bašković M., Markić J., Pogorelić Z. (2025). Perforated peptic ulcers in children: A systematic review. BMC Pediatr..

[4] Hudnall A., Bardes J.M., Coleman K., Stout C., Regier D., Balise S., Borgstrom D., Grabo D. (2022). The surgical management of complicated peptic ulcer disease: An EAST video presentation. J. Trauma. Acute Care Surg..

[5] Chung K.T., Shelat V.G. (2017). Perforated peptic ulcer-an update. World J. Gastrointest. Surg..

[6] Pereira A., Santos Sousa H., Goncalves D., Lima da Costa E., Costa Pinho A., Barbosa E., Barbosa J. (2021). Surgery for Perforated Peptic Ulcer: Is. Laparoscopy a New Paradigm?. Minim. Invasive Surg..

[7] Pogorelić Z. (2022). Advances and future challenges of minimally invasive surgery in children. Children.

[8] Chan K.S., Wang Y.L., Chan X.W., Shelat V.G. (2021). Outcomes of omental patch repair in large or giant perforated peptic ulcer are comparable to gastrectomy. Eur. J. Trauma. Emerg. Surg..

[9] Manfredini R., De Giorgio R., Smolensky M.H., Boari B., Salmi R., Fabbri D., Contato E., Serra M., Barbara G., Stanghellini V. (2010). Seasonal pattern of peptic ulcer hospitalizations: Analysis of the hospital discharge data of the Emilia-Romagna region of Italy. BMC Gastroenterol..

[10] Guo C.G., Tian L., Zhang F., Cheung K.S., Leung W.K. (2021). Associations of seasonal variations and meteorological parameters with incidences of upper and lower gastrointestinal bleeding. J. Gastroenterol. Hepatol..

[11] Li B., Huang W., Chen P., Chen J., Biviano I., Wang Z. (2022). Effect of ambient temperature on daily hospital admissions for acute pancreatitis in Nanchang, China: A time-series analysis. Int. J. Environ. Health Res..

[12] Yang C., Sugimoto K., Murata Y., Hirata Y., Kamakura Y., Koyama Y., Miyashita Y., Nakama K., Higashisaka K., Harada K. (2020). Molecular mechanisms of Wischnewski spot development on gastric mucosa in fatal hypothermia: An experimental study in rats. Sci. Rep..

[13] Yoon J.Y., Cha J.M., Kim H.I., Kwak M.S. (2021). Seasonal variation of peptic ulcer disease, peptic ulcer bleeding, and acute pancreatitis: A nationwide population-based study using a common data model. Medicine.

[14] Yuan X.G., Xie C., Chen J., Xie Y., Zhang K.H., Lu N.H. (2015). Seasonal changes in gastric mucosal factors associated with peptic ulcer bleeding. Exp. Ther. Med..

[15] Lai Y.C., Chen Y.H., Chen C.A., Ho C.H., Wu Y.C., Wang J.J., Weng S.F., Kao Y. (2024). Seasonal variations in peptic ulcer disease incidence in Taiwan, a country spanning both tropical and subtropical regions: A real-world database analysis. BMJ Open Gastroenterol..

[16] Sonnenberg A., Wasserman I.H., Jacobsen S.J. (1992). Monthly variation of hospital admission and mortality of peptic ulcer disease: A reappraisal of ulcer periodicity. Gastroenterology.

[17] Savarino V., Mela G.S., Zentilin P., Lapertosa G., Cutela P., Mele M.R., Mansi C., Dallorto E., Vassallo A., Celle G. (1996). Are duodenal ulcer seasonal fluctuations paralleled by seasonal changes in 24-hour gastric acidity and Helicobacter pylori infection?. J. Clin. Gastroenterol..

[18] Christensen A., Hansen C.P., Thagaard C., Lanng C. (1988). Seasonal periodicity of perforated gastric ulcer. Dan. Med. Bull..

[19] Wysocki A., Budzynski P., Kulawik J., Drozdz W. (2011). Changes in the localization of perforated peptic ulcer and its relation to gender and age of the patients throughout the last 45 years. World J. Surg..

[20] Anbalakan K., Chua D., Pandya G.J., Shelat V.G. (2015). Five year experience in management of perforated peptic ulcer and validation of common mortality risk prediction models-are existing models sufficient? A retrospective cohort study. Int. J. Surg..

[21] Kocer B., Surmeli S., Solak C., Unal B., Bozkurt B., Yildirim O., Dolapci M., Cengiz O. (2007). Factors affecting mortality and morbidity in patients with peptic ulcer perforation. J. Gastroenterol. Hepatol..

[22] Budzynski P., Pogoda W., Pogodzinski M. (2000). Seasonal variation and influence of atmospheric pressure diurnal fluctuations on occurrence of acute complications in patients with stomach and duodenal ulcer. Przegl. Lek..

[23] Svanes C., Sothern R.B., Sorbye H. (1998). Rhythmic patterns in incidence of peptic ulcer perforation over 5.5 decades in Norway. Chronobiol. Int..

[24] Adler J., Ingram D., House T. (1984). Perforated peptic ulcer-a seasonal disease?. Aust. N. Z. J. Surg..

[25] Fich A., Goldin E., Zimmerman J., Rachmilewitz D. (1988). Seasonal variations in the frequency of endoscopically diagnosed duodenal ulcer in Israel. J. Clin. Gastroenterol..

[26] Kanotra R., Ahmed M., Patel N., Thakkar B., Solanki S., Tareen S., Fasullo M.J., Kesavan M., Nalluri N., Khan A. (2016). Seasonal variations and trends in hospitalization for peptic ulcer disease in the United States: A 12-year analysis of the nationwide inpatient sample. Cureus.

[27] Yaratha K., Talemal L., Monahan B.V., Yu D., Lu X., Poggio J.L. (2023). Seasonal and geographic variation in peptic ulcer disease and associated complications in the United States of America. J. Res. Health Sci..

[28] Nomura T., Ohkusa T., Araki A., Chuganji Y., Momoi M., Takashimizu I., Watanabe M. (2001). Influence of climatic factors in the incidence of upper gastrointestinal bleeding. J. Gastroenterol. Hepatol..

[29] Xirasagar S., Lin H.C., Chen C.S. (2007). Role of meteorological factors in duodenal ulcer seasonality: A nation-wide, population-based study. J. Gen. Intern. Med..

[30] Fares A. (2013). Global patterns of seasonal variation in gastrointestinal diseases. J. Postgrad. Med..

[31] Ko J.K., Cho C.H. (2000). Alcohol drinking and cigarette smoking: A “partner” for gastric ulceration. Chin. Med. J..

[32] Kortas D.Y., Haas L.S., Simpson W.G., Nickl N.J., Gates L.K. (2001). Mallory-Weiss tear: Predisposing factors and predictors of a complicated course. Am. J. Gastroenterol..

[33] Yawar B., Marzouk A.M., Ali H., Ghorab T.M., Asim A., Bahli Z., Abousamra M., Diab A., Abdulrahman H., Asim A.E. (2021). Seasonal Variation of Presentation of Perforated Peptic Ulcer Disease: An Overview of Patient Demographics, Management and Outcomes. Cureus.

[34] Liu Y., Xiao Z., Ye K., Xu L., Zhang Y. (2022). Smoking, alcohol consumption, diabetes, body mass index, and peptic ulcer risk: A two-sample Mendelian randomization study. Front. Genet..

[35] Canoy D.S., Hart A.R., Todd C.J. (2002). Epidemiology of duodenal ulcer perforation: A study on hospital admissions in Norfolk, United Kingdom. Dig. Liver Dis..

